# Plasmon Assisted Highly Efficient Visible Light Catalytic CO_2_ Reduction Over the Noble Metal Decorated Sr-Incorporated g-C_3_N_4_

**DOI:** 10.1007/s40820-021-00736-x

**Published:** 2021-10-15

**Authors:** Muhammad Humayun, Habib Ullah, Lang Shu, Xiang Ao, Asif Ali Tahir, Chungdong Wang, Wei Luo

**Affiliations:** 1 School of Optical and Electronic Information, Wuhan National Laboratory for Optoelectronics, Engineering Research Center for Functional Ceramics of the Ministry of Education, Huazhong University of Science and Technology, Wuhan, 430074 People’s Republic of China; 2 Environment and Sustainability Institute, University of Exeter, Cornwall, Penryn, TR10 9FE UK

**Keywords:** g-C_3_N_4_, Sr-incorporation, Noble metal deposition, Density functional theory, Energy applications

## Abstract

The photocatalytic performance of g-C_3_N_4_ for CO_2_ conversion is still inadequate by several shortfalls including the instability, insufficient solar light absorption and rapid charge carrier’s recombination rate. To solve these problems, herein, noble metals (Pt and Au) decorated Sr-incorporated g-C_3_N_4_ photocatalysts are fabricated via the simple calcination and photo-deposition methods. The Sr-incorporation remarkably reduced the g-C_3_N_4_ band gap from 2.7 to 2.54 eV, as evidenced by the UV–visible absorption spectra and the density functional theory results. The CO_2_ conversion performance of the catalysts was evaluated under visible light irradiation. The Pt/0.15Sr-CN sample produced 48.55 and 74.54 µmol h^−1^ g^−1^ of CH_4_ and CO, respectively. These amounts are far greater than that produced by the Au/0.15Sr-CN, 0.15Sr-CN, and CN samples. A high quantum efficiency of 2.92% is predicted for the Pt/0.15Sr-CN sample. Further, the stability of the photocatalyst is confirmed via the photocatalytic recyclable test. The improved CO_2_ conversion performance of the catalyst is accredited to the promoted light absorption and remarkably enhanced charge separation via the Sr-incorporated mid gap states and the localized surface plasmon resonance effect induced by noble metal nanoparticles. This work will provide a new approach for promoting the catalytic efficiency of g-C_3_N_4_ for efficient solar fuel production.

**Supplementary Information:**

The online version contains supplementary material available at 10.1007/s40820-021-00736-x.

## Highlights

- Noble metals (Pt, Au) decorated Sr-incorporated g-C_3_N_4_ photocatalysts are fabricated via facile calcination and photo-deposition methods.
- The optical absorption and charge separation properties of the photocatalysts are remarkably improved and the Pt/0.15Sr-CN photocatalyst exhibited excellent activity for CO_2_ conversion.
- A quantum efficiency of 2.92% is predicted for CO_2_ reduction over the Pt/0.15Sr-CN photocatalyst at 420 nm wavelength, which is accredited to the improved optical absorption and enhanced charge separation via Sr-incorporation and the surface plasmon resonance effect of noble metal nanoparticles.

**Supplementary Information:**

The online version contains supplementary material available at 10.1007/s40820-021-00736-x.

## Graphical abstract

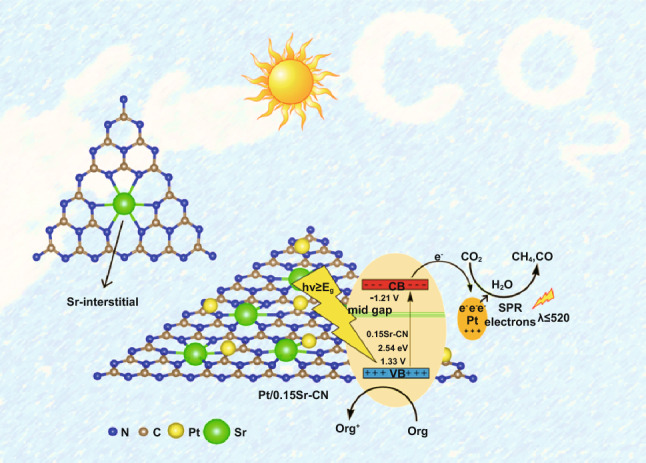

## Introduction

The energy shortage driven by the rapid depletion of fossil fuels and the significant rise in atmospheric CO_2_ concentration are the main challenges facing mankind these days [1–4]. This dramatic increase in CO_2_ concentration (i.e., from 280 to 410 parts per million (ppm)) has been considered as one of the major contributors to the greenhouse effect that seriously smashed the natural carbon balance and provoked global warming [5, 6]. According to the recent reports [7, 8], the current CO_2_ concentration in the atmosphere exceeded 415 ppm, which is expected to raise up to 500 ppm by the end of 2030. In order to stabilize global warming, 70–80% of the CO_2_ should be reduced to keep the threshold below 2 °C. In fact, the highly positive Gibbs free energy change for CO_2_ conversion to CH_4_ (i.e., 1135 kJ mol^−1^) makes the CO_2_ conversion reactions thermodynamically adverse [9]. Further, due to the extremely stable and unreactive nature of CO_2_ molecules, its conversion to value added fuels is a challenging problem that requires high energy input [10]. To overcome these issues, traditional-biological and electro-catalytic techniques are broadly explored, but they always exhibit several drawbacks such as instability, weak mechanical strength, electrode corrosion, and catalyst poisoning. Hence, it is of immense interest to develop alternative strategies to effectively reduce CO_2_ into value added products [11].

Fortunately, photocatalysis with the aid of semiconductor photocatalysts could be employed to facilitate these reduction reactions. However, suitable redox potentials and strong solar light absorption properties are necessary for efficient photocatalytic reactions, which most of the semiconductors do not possess [12, 13]. So far, TiO_2_ has gained considerable attention in photocatalysis owing to its low cost, high stability, high performance, and nontoxicity. Yet, the practical applications of TiO_2_ are still inadequate owing to its large band gap (E_g_ = 3.2 eV) that could utilize only 4% (UV) of the total solar energy. In order to improve its light absorption and charge separation functionalities, various modifications tactics are generally employed [14, 15]. Nevertheless, the modifications made are far from the idea goal of harvesting light absorption and charge transport.

Graphite carbon nitride (g-C_3_N_4_) as a metal free organic polymeric semiconductor received increasing attention owing to its low cost, natural abundance, and flexible electronic structure. The g-C_3_N_4_ has been regarded as a visible light active photocatalytic because of its absorption up to 460 nm. Since, the g-C_3_N_4_ is composed of aromatic heterocyclic rings of C − N bonds. Hence, it is thermally stable up to 600 °C in air atmosphere. Besides, it exhibits high chemical stability in various solvents including alcohols, toluene, water, diethyl ether, N, N-dimethylformamide, glacial acetic acid, and 0.1 M aqueous solution of sodium hydroxide due to the existence of strong van der Waal’s forces in its layered structure [16, 17]. Since the first report on g-C_3_N_4_-based photocatalysis in 2009, it has been widely employed in CO_2_ conversion and water splitting [18, 19]. Yet, the photocatalytic efficiency of g-C_3_N_4_ still has immense gap to improve, owing to its insufficient light harvesting, rapid charge recombination, and small surface area [20]. To prevail over these shortfalls, a number of strategies including the metal/non-metal doping, surface defects and vacancies generation, noble metal deposition, and semiconductors coupling have been developed [21–23]. Among various modification tactics, elemental doping and heterojunctions construction are usually employed to improve the light harvesting ability, charge carrier’s separation, and catalytic efficiency of g-C_3_N_4_. For example, Zhu et al. [24] fabricated non-metal S-doped g-C_3_N_4_ via hydrothermal route. The photocatalyst showed high performance for CO_2_ conversion to CO, which was accredited to the enhanced light absorption owing to the decrease in band gap and significant electron–hole pairs separation. Non-metal P-doped g-C_3_N_4_ photocatalyst also exhibited high efficiency for CO_2_ reduction to CO under visible light as reported by Huang and his co-workers [25]. According to Dong et al. [26], Mg-doped g-C_3_N_4_ catalyst fabricated via a facile one-pot method displayed high performance for CO_2_ photoreduction due to the extended light absorption and improved charge separation efficiency via the introduced mid gap states of Mg. Likewise, Tang et al. [27] introduced Mg in g-C_3_N_4_ via an in-situ hydrothermal technique that displayed enhanced activity for CO_2_ photoreduction as a result of the improved light response and charge separation.

Recently, strontium (Sr) an alkaline earth metal with electronic configuration [Kr] 5 s^2^, received tremendous attention in photocatalysis owing to its special physical and chemical properties. Thus, Sr and its compounds have been widely utilized in CO_2_ capture and storage, hydrogen storage, and catalytic CO_2_ reduction reactions [28–30]. The incorporation of Sr atoms into the g-C_3_N_4_ framework reduces its band gap, thereby remarkably enhances its visible light response and catalytic performance [31, 32]. Thus, it is of great importance to incorporate Sr atoms into g-C_3_N_4_ to extend its visible light response for effective CO_2_ conversion.

Besides elemental doping, the noble metal nanoparticles such as Au and Pt, deposition over the g-C_3_N_4_ also have a critical role in improvement of the light absorption and charge carrier’s separation as a result of the localized surface plasmon resonance (SPR) effect induced by the noble metal nanoparticles. Thus, employing the SPR effect, noble metal nanoparticles deposition is another viable option as a modifier for semiconductors to extend their visible light response for efficient photocatalysis. In addition, the noble metal nanoparticles have strong resistance to corrosion and oxidation under moist condition [33, 34].

For instance, Li et al. [35] prepared Au-deposited g-C_3_N_4_ photocatalyst via the NaBH_4_-reduction technique that showed significant CO_2_ photoreduction to CH_4_. This was accredited to the improved light absorption and promoted charge separation via the SPR effect of Au nanoparticles. According to Gao et al. [10], Pd and Pt decorated g-C_3_N_4_ displayed high performance for CO_2_ photoreduction, which was accredited to the improved light absorption and charge separation via the SPR effect of Pd and Pt nanoparticles.

Though, there are abundant publications on the modification of g-C_3_N_4_, but the charge transfer mechanisms related to activity enhancement still need further investigations. Herein, we designed noble metal (Pt, Au) decorated Sr-incorporated g-C_3_N_4_ photocatalysts and employed in photocatalysis for efficient CO_2_ conversion. We carried out a detail experimental and theoretical investigation, which led us to the conclusion that interstitial site doping is the most favorable in case of Sr-incorporated g-C_3_N_4_. Further, we utilized the SPR effect of noble metal nanoparticles (i.e., Pt and Au) and promoted the surface redox activity of the optimized 0.15Sr-CN photocatalyst. Worth noting, when the catalysts were irradiated under visible light, charge carriers were produced. The excited high energy electrons of 0.15Sr-CN were migrated to the Pt and Au surface and effectively reduced CO_2_ molecules to CH_4_ and CO products. Accordingly, the superior catalytic activity of the CN is accredited to the promoted light absorption via the decrease in band gap after Sr-incorporation and enhanced charge separation and transfer and surface redox ability via the localized SPR effect of noble metal nanoparticles. This work would offer new strategies to promote charge carrier’s separation and catalytic performance of g-C_3_N_4_ for prospective energy applications.

## Experimental Section

### Precursor Materials and Synthesis

The chemical, precursors, and reagents were purchased from Sinopharm-Chemical Reagent Co. Ltd. (Shanghai, China) and used as such.

The g-C_3_N_4_ was simply prepared by annealing 10 g dicyandiamide precursor in air environment at 550 °C (5 °C min^−1^) for 2 h. The yellow product was crushed into fine powder and again annealed at 550 °C for 2 h to achieve sheet-like structure by further condensation of polymer.

To prepare different amount Sr-incorporated g-C_3_N_4_ samples, *x* amount of strontium nitrate (Sr(NO_3_)_2_) salt (*x* = 0.05, 0.10, 0.15, and 0.2 g) was mixed with every 10 g of dicyandiamide precursor. The mixtures were crushed into fine powder in agate-mortar and then transferred to a ceramic crucible partially covered with a lid. The mixtures were annealed at 550 °C (5 °C min^−1^) for 2 h. After cooling down naturally, the powders were re-calcined at the same temperature for 2 h to obtain sheet-like Sr-incorporated g-C_3_N_4_. The products were grinded into fine powder.

The Pt/0.15Sr-CN and Au/0.15Sr-CN photocatalysts were fabricated via simple photo-deposition process. For both samples, 1 g of the optimized 0.15Sr-CN sample powder was dispersed into 80 mL of absolute methanol in two separate beakers. Then appropriate amount of HAuCl_4_·4H_2_O solution prepared in de-ionized water (containing 2% by mass of Au) was added to one beaker, and proper amount of H_2_PtCl_6_·xH_2_O solution (containing 2% by mass of Pt) was added to another beaker. Each flask containing the sample and corresponding Pt and Au solution was well covered, and then N_2_ gas was bubbled through them for half an hour to generate inert atmosphere for photoreduction of Pt and Au nanoparticles thereby removing the dissolved O_2_. Simultaneously, the samples were irradiated with 300 W Xe-lamp (wavelength range of 200–400 nm), under dynamic stirring for 2 h. Finally, the light treated catalysts were centrifuged, continuously washed with de-ionized water and subsequently vacuum dried at 65 °C. The samples were labeled as Pt/0.15Sr-CN and Au/0.15Sr-CN.

### Materials Characterization

The x’pert3 PANalytical X-ray diffraction-(XRD) spectrometer (Netherlands) was used for structural characterization. The PerkinElmerLambda-35 UV–Vis spectrophotometer-(USA) was employed for absorption spectra measurements. The Geminisem-(300–7112) scanning electron microscope (SEM) (Germany) was used for SEM micrographs and energy-dispersive X-ray spectroscopy (EDS) mapping analysis. The Talos-(F200x)-FTEM (FEI, Netherland) transmission electron microscope (TEM) was used analysis of micro-images. The DLD-(600 W)-AXIS-ULTRA (Kratos, Japan) X-ray photo-electron spectrometer (XPS) was used for chemical analysis of the samples. The Bruker VERTEX-70 (Germany) Fourier transforms infrared spectrometer (FTIR) was used for functional group analysis. The Raman spectra were recorded with Horiba JobinYvon, LabRAM-HR800 (France). The FP-6500 photoluminescence (PL) spectrometer (Jasco, Japan) was employed for photoluminescence and fluorescence spectra analysis. The electrochemical tests were conducted in a three electrode system with 0.5 M KOH as electrolyte.

### Photocatalytic Conversion of CO_2_

The CO_2_ conversion tests were achieved in an online system connected with gas chromatographs (TCD and FID). The catalyst dose (0.1 g) was dispersed in a 50 mL-H_2_O contained in a 250 mL volume glass cell. The system was irradiated under visible light (cut-off 420 nm) with Xe-lamp (300 W) as a light source. The high purity CO_2_ gas was injected into the system at ambient pressure. The CO_2_/H_2_O system was equilibrated for 1 h. During reaction, the products (CH_4_, CO) concentration was detected at a regular time interval of 1 h.

### Photocatalytic H_2_O Reduction

Photocatalytic H_2_O reduction experiments were performed in an online H_2_O splitting apparatus (Perfect light company, Beijing). For each experiment, 80 mL de-ionized H_2_O and 20 mL CH_3_OH (sacrificial mediator) were mixed in glass cell, and a catalyst dose of 0.1 g was dispersed in it. The system was evacuated for 30 min to create gas-free environment, and the experiments were carried out under visible light irradiations using a Xe-lamp of 300 W. The H_2_ quantity was predicted via an inline gas-chromatograph (CEAULIGH-TCD-7920, N_2_-gas carrier).

### Photodegradation Experiments

For 2,4-DCP degradation experiments, a 100 mL quartz reactor was used, and the catalyst dose (0.2 g) was dispersed in 2,4-DCP solution (80 mL taken from the stock) (concentration = 20 mg L^−1^). About 30 min stirring in dark was made to achieve the adsorption saturation. Subsequently, the solution was kept under visible light irradiation for 2 h (Xe-lamp, 300 W). A proper amount of solution after post catalytic treatment was centrifuged, and the 2,4-DCP concentration was detected via the Lambda-35 spectrophotometer (Perkin-Elmer, USA) at absorption wavelength of 285 nm (specific for 2,4-DCP).

For Rhodamine B (RhB) dye degradation, 20 mg L^−1^ of RhB solution was prepared in advance. The experimental procedure was the same as for that of the 2,4-DCP. However, the RhB concentration was detected at absorption wavelength of 553 nm (specific for RhB).

### Computational Methods

Solid-state density functional theory-(DFT) simulations are achieved via Quantum-ATK, and the visualization is achieved on VNL Version 2019.12 [36]. Both single layer and bulk CN are considered for simulations, where one Sr atom is used as dopant (Sr-CN), and we modeled (i) Sr-CN(interstitial doped), (ii) Sr-CN(C-substituted), and (iii) Sr-CN(N-substituted) as shown in Fig. S7. In addition, we also investigated the effect of different layers of CN over Sr-incorporation, and the optimized models are shown in Fig. S8. Actually, we tried many methods such as Generalized-gradient approximation-(GGA)/PerdewBurkeErnzerhof-(PBE), meta-GGA, spin polarized GGA (SGGA), and GGA + U. It is found that GGA can accurately perform the optimization. The GGA via the PBE exchange correlation-functional and double-Zeta Polarized-(DZP) basis set is employed for energy and structural-optimization owing to its advantage over the hybrid pseudo-potentials [37]. We have used linear-combination of atomic-orbital’s (LCAO) technique for Sr, C, N, and H atoms. A 7 × 7 × 7 Monkhorst-Packed k-grid and cut-off energy of 1200 eV is employed for bulk CN and its doped species whereas a 7 × 7 × 1 k-point-mesh is employed for single layer CN. The band-structure calculations were achieved on meta-GGA with TB09LDA-functional which has the ability to precisely reproduce the experimentally obtained band gaps. Recently, Tran and Blaha reported that the precision of this method is because of utilizing the local-density ρ(r)(as in LDA), the density gradient ∇ρ(r)(as in GGA), and the kinetic energy–density τ(r) [38]. In this work, the c-factor of Tran and Blaha XC functional equation was fitted to excellently reproduce the experimentally obtained band gaps [38]. The Partial-density of states-(PDOS) and the electrostatic-potential maps are also calculated. The DFT-occupied and unoccupied-DOS are considered as the VB and CB boundaries, respectively [39].

## Results & Discussion

### Structural Morphology and Chemical Composition

As clear from the XRD results (Figs. S1a and 1a), g-C_3_N_4_ (CN) exhibits two apparent peaks at 2θ = 13.1° and 27.4°, analogous to the in-plane structural-packing motifs (100-plane) and interlayer stacking of the conjugated aromatic-structure (002-plane), respectively. The XRD pattern of pure CN is well matched with the JCPDS No. 87–1522 [32]. The XRD patterns of *x*Sr-CN samples do not exhibit any impurity peaks. However, the apparent peak of all samples at 27.4^○^ is slightly shifted toward lower degree, signifying that the incorporation of Sr reduces the layer distance of CN. This may be due to the fact that the interstitial Sr might weaken the repulsive force among the adjacent layers or attracted the negative charge N atoms in the (002) lattice plane. So far, the interlayer spacing of CN (3.16 Å) is larger than the diameter of Sr-cation (2.24 Å), and 4d orbital of Sr-cation is empty. In addition, both C and N atoms have lone pair electrons, hence, Sr-cation would form coordination bond with C or N by accepting their lone pair electrons, thereby replacing C or N atoms. Thus, Sr-cation may have multiple site doping possibilities in the CN. The XRD patterns of Au/0.15Sr-CN sample exhibit extra peaks at 38.2^○^, 44.3^○^, 64.4^○^, and 77.6^○^ corresponding to the Au (111), (200), (220), and (311) planes, respectively [35]. This demonstrates that metallic Au was successfully photodeposited onto the surface of CN. Worth noting, the XRD patterns of Pt/0.15Sr-CN sample do not exhibit any diffraction peak of metallic Pt. This might be accredited to the fact that Pt is uniformly dispersed onto the photocatalyst surface [40]. In addition, no shift in the diffraction peaks of Au/0.15Sr-CN and Pt/0.15Sr-CN samples can be observed. These observations suggest that photo-deposition of Au and Pt has no effect on the structure of 0.15Sr-CN catalyst.

**Fig. 1:**
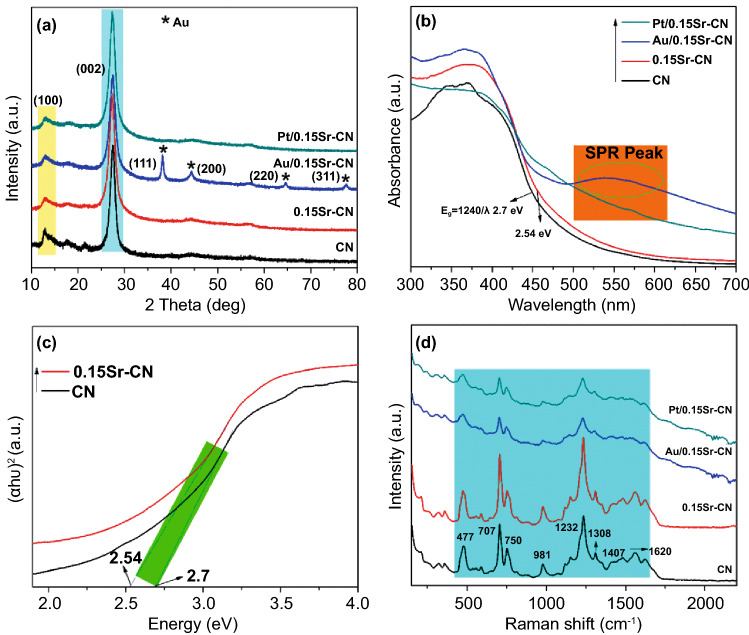
**a** XRD patterns and **b** UV–visible absorption spectra of CN, 0.15Sr-CN, Au/0.15Sr-CN, and Pt/0.15Sr-CN. **c** Estimated band gaps of CN and 0.15Sr-CN and **d** Raman spectra of CN, 0.15Sr-CN, Au/0.15Sr-CN, and Pt/0.15Sr-CN

As widely accepted, the optical behavior of photocatalysts can remarkably affect the photocatalytic performance due to the excitation of electrons that depends on light absorption. Thus, optical performance of the catalysts was analyzed via the UV–vis absorption spectroscopy, and the spectra are displayed in Figs. 1b and S1b. The absorption edge of pristine CN could be found at nearly 470 nm, analogous to the band gap of 2.7 eV, as predicted by the Kubelka–Munk equation and the Tauc plots ((*αhν*)^2^ *vs. hν*)) (Figs. 1c and S1c). This absorption peak corresponds to the band-to-band transitions in CN. Compared with pristine CN, the absorption edges of *x*Sr-CN photocatalysts are slightly red-shifted with the increase in Sr amount (Fig. S1b), signifying that the incorporation of Sr can extend the light absorption of CN. The Sr-incorporation has reduced the CN band gap from 2.7 to 2.54 eV, as predicted by Kubelka–Munk equation and the Tauc plots (Fig. S1b). It is noteworthy, the absorption edges of Au/0.15Sr-CN and Pt/0.15Sr-CN samples (Fig. S1d, e) didn’t change, indicating that Au and Pt are not doped into the 0.15Sr-CN framework, but instead deposited onto its surface. The absorption spectrum of Au/0.15Sr-CN (Fig. 1b) sample exhibits an extra broad peak centering at 550 nm. This extra band is accredited to the SPR effect of Au nanoparticles. However, SPR peak of the Pt/0.15Sr-CN sample is relatively weak, which may be due to the fact that Pt nanoparticles are highly dispersed onto the surface of 0.15Sr-CN photocatalyst. This reveals that the electronic structure of 0.15Sr-CN catalysts is greatly modified via the photo-deposition of Au and Pt nanoparticles due to the strong interaction between 0.15Sr-CN and the noble metal nanoparticles.

Raman spectroscopy measurements were performed to probe the crystalline-structure of CN, *x*Sr-CN, and the Pt/Au-deposited 0.15Sr-CN samples. Raman technique is highly sensitive to variation in the lattice symmetry. As displayed in Figs. 1d and S1f, the Raman spectrum of CN exhibit characteristic peaks in the range of 450–1620 cm^−1^, which are accredited to the graphitic carbon nitride motif. The peaks at 750, 981, and 1232 cm^−1^ are accredited to the stretching-vibration mode of aromatic CN heterocycles in melem. The Raman peak at 707 cm^−1^ is accredited to the breathing vibration modes of the s-triazine ring. Besides, the presence of peaks at 1308 (D-band) and 1556 cm^−1^ (G-band) confirms the formation of graphitic carbon nitride [41]. Interestingly, these characteristic peaks also appeared in the *x*Sr-CN samples, suggesting that Sr-incorporation has not damaged the skeleton of CN. However, the characteristic Raman peaks of *x*Sr-CN samples are slightly blue shifted, and their intensities are gradually enhanced with the increase in Sr-content. Worth noting, after photo-deposition of Au and Pt nanoparticles (Fig. 1d), the characteristic peaks intensity of 0.15Sr-CN remarkably reduced, which may be due to the fact that Au and Pt nanoparticles result into the scattering mode of CN network [42].

The photocatalysts morphology was explored via the SEM technique as shown in Figs. S2 and S3. The pristine CN exhibits distinctive sheet-like morphology (Fig. S2a). Nevertheless, it is hard to identify a solitary nanosheet because of the rigorous restacking of sheets [43]. Obviously, the Sr-incorporation didn’t change the morphology of CN (Fig. S2b-e). As displayed in Fig. S3a, b, the SEM micrographs of Au/0.15Sr-CN and Pt/0.15Sr-CN samples reveal the presence of small Au and Pt nanoparticles dispersed onto the 0.15Sr-CN catalyst surface. The EDS elemental-mapping images of CN catalyst (Fig. S4a-c) clarifies the well distribution of C (red color) and N (green color) elements. The EDS elemental mapping images of 0.15Sr-CN (Fig. S5a-d) show the distribution of C (green color), N (cyan color), and Sr (red color) elements. Further, the EDX spectrum of CN specifies the relevant peaks of C and N elements with their respective atomic percentage composition of 40.57% and 59.43% (Fig. S2f). Moreover, the EDX spectra of *x*Sr-CN samples (Fig. S2g-j) confirm the peaks of C, N, and Sr elements, and the atomic percentage composition of each element is given as inset.

**Fig. 2:**
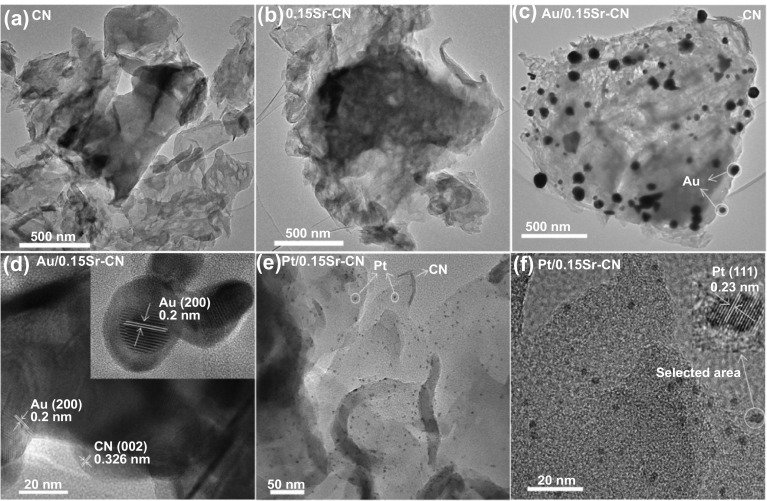
TEM micrographs of **a** CN, **b** 0.15Sr-CN and **c** of Au/0.15Sr-CN. HRTEM micrograph of **d** Au/0.15Sr-CN sample. **e** TEM micrograph and **f** HRTEM micrograph of Pt/0.15Sr-CN

The catalysts morphology was further investigated by TEM technique as shown in Fig. 2. The TEM micrographs in Fig. 2a, b display that pristine CN and 0.15Sr-CN samples exhibit sheet-like morphology, and the Sr-incorporation didn’t influence its morphology. The TEM image of Au/0.15Sr-CN sample (Fig. 2c) reveals the well dispersed Au nanoparticles (size = 10–15 nm) onto the surface of 0.15Sr-CN sample. The HRTEM micrograph of Au/0.15Sr-CN (Fig. 2d) reveals the distinct lattice fringes of Au (200) and CN (002) with spacing of 0.2 and 0.326 nm, respectively. In addition, the TEM micrograph of Pt/0.15Sr-CN sample (Fig. 2e) reveals the highly dispersed small sized (1–3 nm) Pt nanoparticles onto the surface of 0.15Sr-CN sample. The distinctive lattice fringes of Pt (111) with spacing 0.23 nm can be observed from the HRTEM micrograph of Pt/0.15Sr-CN (Fig. 2f).

**Fig. 3:**
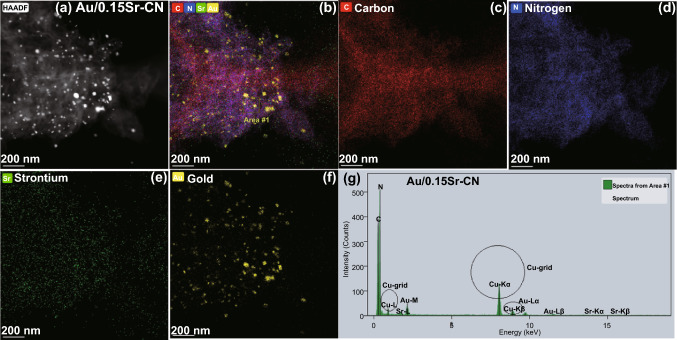
TEM mappings of **a, b** Au/0.15Sr-CN, **c** carbon element, **d** N element, **e** Strontium element, and **f** Gold. **g** EDX spectrum of Au/0.15Sr-CN sample

**Fig. 4:**
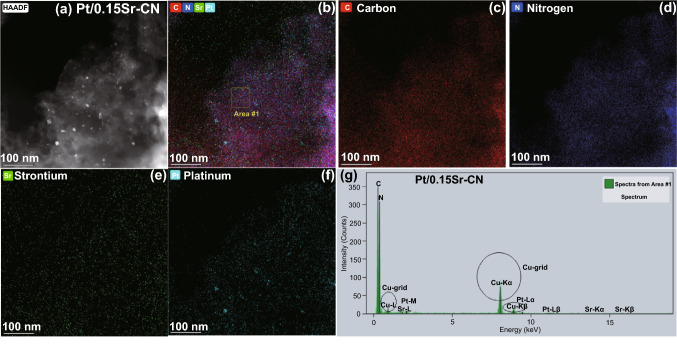
TEM mappings of **a, b** Pt/0.15Sr-CN, **c** carbon element, **d** N element, **e** Strontium element, and **f** Platinum. **g** EDX spectrum of Pt/0.15Sr-CN

The EDS elemental mappings of Au/0.15Sr-CN sample obtained via FTEM (Fig. 3a-f) shows the distribution of C (red color), N (blue color), Sr (green color), and Au (brown color) elements. Further, the EDX spectrum of Au/0.15Sr-CN catalyst (Fig. 3g) clearly demonstrates the peaks of C, N, Sr, and Au elements. In addition, the Cu-grid related peaks can also be observed. The EDS elemental mappings of Pt/0.15Sr-CN sample (Fig. 4a-f) display the uniform distribution of C (red color), N (blue color), Sr (green color), and Pt (cyan color) elements. While, the relevant peaks of C, N, Sr, and Pt can be discerned from the EDX spectrum (Fig. 4g). The TEM analysis further clarify that the Au and Pt nanoparticles are deposited onto the surface of 0.15Sr-CN sample.

**Fig. 5:**
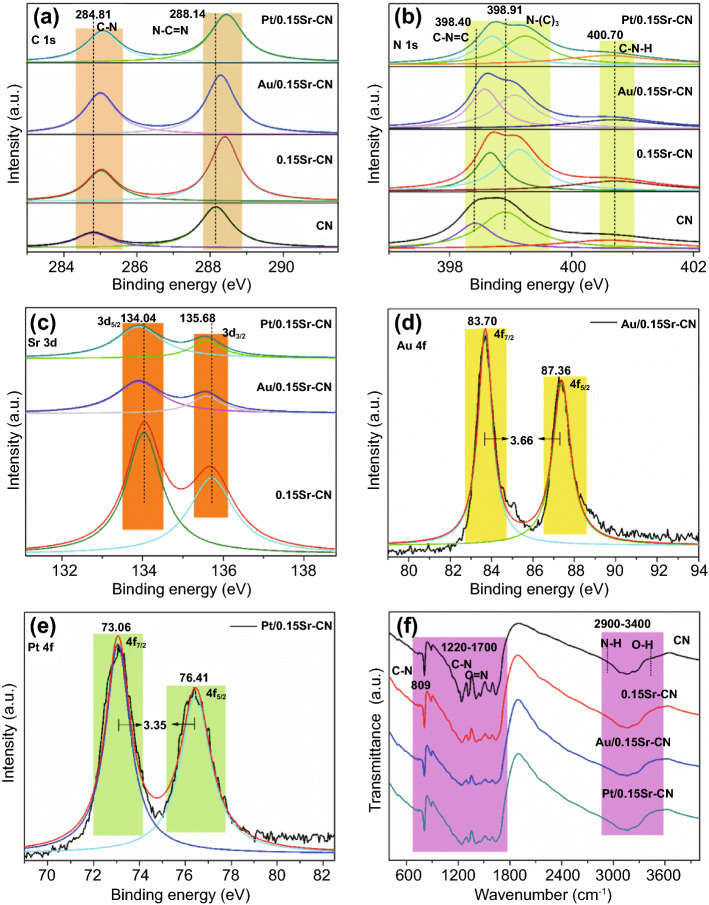
High resolutions of **a** C 1 s-XPS and **b** N 1 s-XPS of CN, 0.15Sr-CN, Au/0.15Sr-CN, and Pt/0.15Sr-CN, **c** Sr 3d XPS of 0.15Sr-CN, Au/0.15Sr-CN, and Pt/0.15Sr-CN, **d** Au 4f XPS of Au/0.15Sr-CN, **e** Pt 4f XPS of Pt/0.15Sr-CN and **f** FTIR spectra of CN, 0.15Sr-CN, Au/0.15Sr-CN, and Pt/0.15Sr-CN

The chemical composition of the catalysts was explored via the XPS technique. The XPS survey spectra of CN, 0.15Sr-CN, Au/0.15Sr-CN, and Pt/0.15Sr-CN catalysts are shown in Fig. S6. The deconvoluted high resolution C 1 s-XPS spectra of CN, 0.15Sr-CN, Au/0.15Sr-CN, and Pt/0.15Sr-CN samples are shown in Fig. 5a. The deconvoluted C 1 s spectrum of CN exhibit two peaks at 284.81 and 288.14 eV, which are accredited to the C − N and N − C = N coordination, respectively [44]. The C 1 s binding energy peaks of 0.15Sr-CN, Au/0.15Sr-CN, and Pt/0.15Sr-CN photocatalysts are slightly red-shifted. The deconvoluted high resolution N1s-XPS spectra of CN, 0.15Sr-CN, Au/0.15Sr-CN, and Pt/0.15Sr-CN samples are provided in Fig. 5b. The deconvoluted N 1 s spectrum of pristine CN exhibits binding energy peaks at 398.40, 398.91, and 400.70 eV. These peaks are ascribed to the *sp*^2^-hybridized aromatic nitrogen (C–N = C), tertiary-nitrogen (N-(C)_3_), and (C–N–H) bonds, respectively [45]. In case of the CN, 0.15Sr-CN, Au/0.15Sr-CN, and Pt/0.15Sr-CN samples, a red-shift in the binding energy peaks can be clearly observed. Interestingly, the binding energy peaks of Sr 3d (Fig. 5c) at 134.04 and 135.68 eV, respectively, corresponding to the Sr 3d_5/2_ and Sr 3d_3/2_ orbital splitting as confirmed by the deconvolution XPS spectra. This reveals that Sr is successfully incorporated into the lattice of g-C_3_N_4_. The Au4f XPS spectrum (Fig. 5d) reveals doublet bands at 83.70 and 87.36 eV, respectively, corresponding to the Au (4f_7/2_ and Au 4f_5/2_) orbitals. As obvious, the difference in the splitting orbital’s energy peaks is 3.66 eV, demonstrating the presence of metallic gold (Au^0^) [46]. Figure 5e shows the high resolution Pt 4f XPS spectrum with two distinct peaks at binding energy values of 73.0 and 76.41 eV, related to the Pt 4f_7/2_ and Pt 4f_5/2_ splitting orbital’s, respectively. The difference in binding energy of these two peaks is 3.35 eV, reflecting the characteristic signal of metallic platinum (Pt^0^) [47]. From FTIR spectra of the samples (Fig. 5f), it is clear that the molecular structure of pristine CN exhibits a broad peak at 2900–3400 cm^−1^, which is accredited to the N─H bonds and the O─H stretching mode. The peaks in the range of 1220 to 1700 cm^−1^ are credited to the C─N and C═N stretching-modes of the aromatic cycles. The pointed peak at 809 cm^−1^ is attributed to the breathing-mode of triazine units [45]. The FTIR spectra of 0.15Sr-CN, Au/0.15Sr-CN and Pt/0.15Sr-CN samples are exactly similar to that of the pristine CN, further confirming that the Sr-incorporation and noble metal nanoparticles deposition do not influence the structure of pristine CN.

### First Principles Study

#### Selection of Doping Site

To understand the binding energy structures and electronic-properties of the samples, DFT simulations were carried out. The favorable doping site for sSr has been predicted from the total free energy and formation energy simulations, as shown in Table S1. The optimized relaxed structures of a single layer CN and its Sr-doped species are given in Fig. S7. In addition, we also investigated the effect of different layers of CN over Sr-incorporation, and the optimized models are shown in Fig. S8. As discussed in the methodology section, Sr is applied to interstitial, N-substituted, and C-substituted sites in the CN. Comparative analysis of the data of Table S1 allows us to conclude that interstitial site is the most favorable attacking site for Sr-incorporated CN, and then C-substitution, which is followed by N substitution with Sr atom. In order to minimize the results, we have limited our discussion to the interstitial site doping, while the data and discussion of C- and N-substituted Sr are provided in the Supporting Information.

#### Electronic Properties

**Fig. 6:**
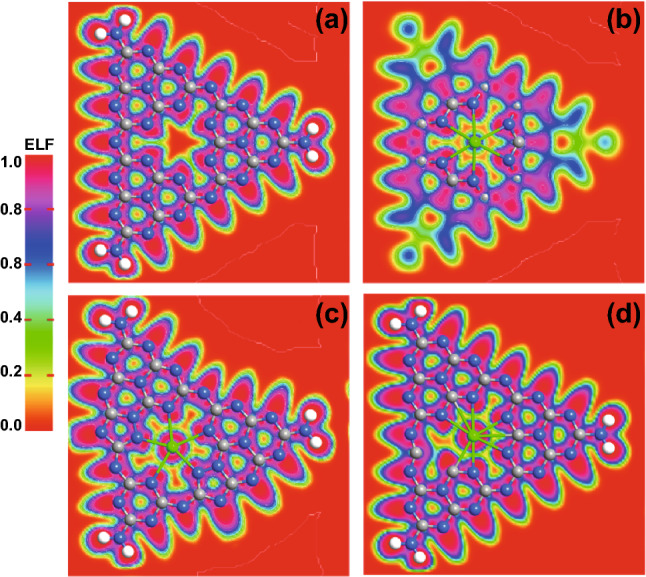
Average electron density difference maps for **a** CN, **b** 0.15Sr-CN (Interstitial site) doping, **c** 0.15Sr-CN (C-substituted) doping, and **d** 0.15Sr-CN (N-substituted) doping

The simulated band-structure of pristine CN is given in Fig. S9a, where the band gap has a nice correlation with our experimental observed one (2.70 eV). This result is also consistent with the previously reported experimental and theoretical reports [16, 48]. Upon interaction with Sr at interstitial site, the band gap reduced to 2.55 eV and the Fermi level moved up to the conduction band (CB) site as shown in Fig. S9b. The reduction in band gap and Fermi level in CB clearly depicts its increase in metallicity compared to the pristine CN. Again, this reduction in band gap, upon interstitial doping of Sr is consistent with our observed experimental data (i.e., 2.54 eV). On the other hand, Sr substituted C and N sites have some extra inter-bands within the VB and CB, as shown in Fig. S9c, d, respectively. It suggests that the existence of these extra bands near the VB states are because of the dangling atoms or instability of the structures resulted from the substitution of Sr either at C or N sites. Another possible reason is the large atomic radius of Sr atom compared to both of the C and N atoms, which distort the resulting structure. This distortion can also be seen from the electron density difference maps, as shown in Fig. 6. So, again, it is confirmed that interstitial position is a favorable doping site compared to that of the C and N substitution sites.

Generally, the partial-density of state (PDOS) contributes to the VB maxima (VBM) and CB maxima (CBM). The comparative PDOS plots of CN and its Sr-doped species are given in Fig. S10. From Fig. S10a, we can analyze that the VBM and CBM of pristine CN consist of p-orbital of N and C atoms, respectively, and are located at − 7.21 and − 4.51 eV versus vacuum. On the other hand, the VBM of 0.15Sr-CN (interstitial site doping) is mainly composed of p-orbital of N atoms; while its CBM is constituted from the 5d orbital’s of the Sr atoms, as shown in Fig. S10b. In addition, it is observed that PDOS of the N atoms in the 0.15Sr-CN (interstitial site doping) system, near the VBM is not hybridized with orbital’s of C and H atoms, leading to lower density of states. While, the density of states of CBM is much increased due to the strong hybridization of anti-boding orbitals of C, N, H, and Sr. This overall strong hybridization at CBM enhanced the overall surface redox ability, stability, and catalytic activity of the 0.15Sr-CN (interstitial site doping) system. The PDOS analysis of 0.15Sr-CN (C-substituted) doping and 0.15Sr-CN (N-substituted) doping systems indicate somehow similar type of hybridization at their VBM and CBM but arise some inter-bands as depicted in Fig. S10c, d. These isolated flat bands or inter-bands are due to un-hybridized orbitals of Sr atom. As discussed above, Sr has larger atomic radii and higher valence state, which does not good fit in place of either C or N atoms. So, we can say that these extra bands are due to dangling or unhybridized orbitals of Sr. Thus, it can be concluded that the Sr-CN (interstitial site doping) has strong overlapping and liable for proficient photocatalytic processes. These statements strongly corroborate our experimental study as well. The Schematic illustration of the band edge positions along with work functions is shown in Fig. S11. The band-alignment of the explored species was predicted via the Eq. (1):

$$\Phi \, = E_{vac} {-} \, E_{F}$$

Finally, the simulated work functions of CN and Sr-CN systems such as interstitial site, C-substituted site, and N-substituted site are simulated which are 5.84, 4.51, 5.51, and 4.73 eV, respectively (Table S2). The simulated work function suggests that Sr-incorporation at the interstitial site significantly decreased the work function which can consequently improve the photo-induced charge carrier’s transport across the interfaces of catalysts. The work functions of 0.15Sr-CN (C-substituted site) and 0.15Sr-CN (N-substituted site) doping were also decreased from 5.84 eV (for CN) to 5.51 and 4.73 eV, respectively. But again, this decrement is comparatively lower than that of the 0.15Sr-CN (interstitial site) doping.

#### Charge Transfer Analysis

**Fig. 7:**
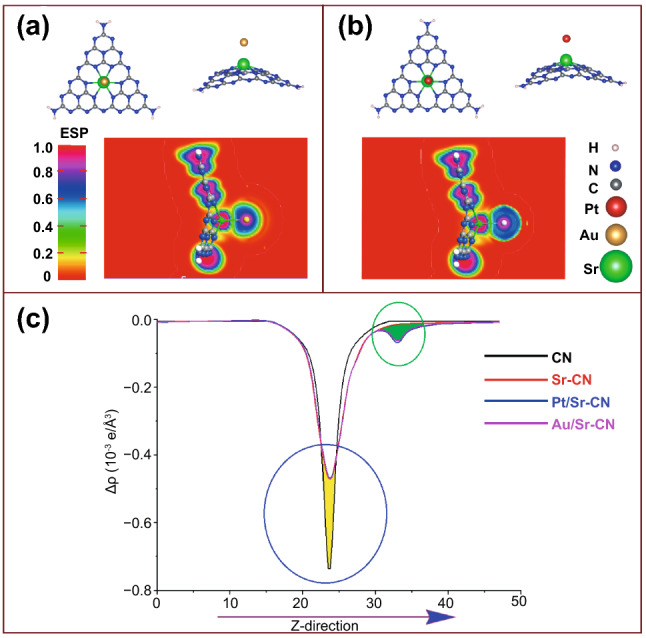
The optimized geometric structure of Au/0.15Sr-CN along with side view and ESP (**a**), the optimized relaxed structure of Pt/0.15Sr-CN with side view and ESP (**b**), and combined 2D electrostatic-potential map of pristine single layer CN, 0.15Sr-CN, Pt/0.15Sr-CN, and Au/0.15Sr-CN catalysts (**c**). In plot (c), the yellow shaded area denotes donation while green shaded area represents electron accumulation

To validate the effect of Pt and Au nanoparticles deposited onto the 0.15Sr-CN catalyst surface, we optimized their geometries as depicted in Fig. 7. As clear from Fig. 7a, b, both Pt and Au atoms are interacted with the Sr atom of 0.15Sr-CN catalyst. The electrostatic-potential map and 2D plots (Fig. 7c) of Pt/0.15Sr-CN and Au/0.15Sr-CN catalysts clearly reveal the electron transfer from Sr to either Pt or Au atoms. This electron-transferring phenomenon can be visualized from the ESP maps (Fig. S12) and 2D plots of Pt/0.15Sr-CN and Au/0.15Sr-CN, where Sr has withdrawn electron cloud density from CN and then this charge is transferred to either Pt or Au atoms. Consequently, both Pt and Au become more electronegative and cause efficient reduction of CO_2_ and H_2_O.

### Photo-Induced Charges

**Fig. 8:**
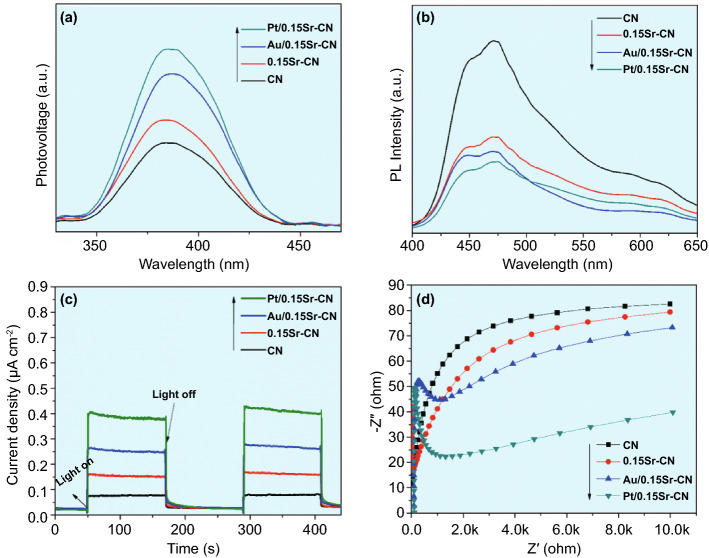
**a** Surface photovoltage responses, **b** photoluminescence responses, **c** photo-electrochemical *I-t* curves, and **d** electrochemical impedance spectra of CN, 0.15Sr-CN, Au/0.15Sr-CN, and Pt/0.15Sr-CN

To explore the charge separation behavior, surface photovoltage (SPV) analysis of the CN, 0.15Sr-CN, Au/0.15Sr-CN, and Pt/0.15Sr-CN was carried out as displayed in Fig. 8a. The SPV signal usually reveals the electronic transition from valance band to the conduction band. Thus, stronger the SPV signal, higher will be the charge separation [21, 49]. As obvious, the SPV signal intensity of the samples is in the order of Pt/0.15Sr-CN > Au/0.15Sr-CN > 0.15Sr-CN > CN. The SPV results validate best charge separation in the Pt/0.15Sr-CN sample. For further evidence, the photoluminescence (PL) spectra of the CN, *x*Sr-CN, Au/0.15Sr-CN, and Pt/0.15Sr-CN samples were also obtained as displayed in Figs. S13a and 8b.

The PL gives us information regarding surface defects, oxygen vacancies and charge transport in semiconductors. Normally, the strong PL signals always reveal high charge recombination rate [50, 51]. Worth noting, the PL intensity signal of *x*Sr-CN samples is extremely decreased especially that of the 0.15Sr-CN one. The PL intensity of pristine CN, optimized 0.15Sr-CN, Au/0.15Sr-CN, and Pt/0.15Sr-CN samples is in the order of CN > 0.15Sr-CN > Au/0.15Sr-CN > Pt/0.15Sr-CN. This further validates the improved charge separation in the Pt/0.15Sr-CN photocatalyst. The photocurrent (*I-V*)-curves of the CN, 0.15Sr-CN, Au/0.15Sr-CN, and Pt/0.15Sr-CN samples (Fig. S13b) reveal that charge separation in Pt/0.15Sr-CN is much superior. The photocurrent density (*I-t*) curves of the CN, 0.15Sr-CN, Au/0.15Sr-CN, and Pt/0.15Sr-CN samples (Fig. 8c) were obtained to further reveal charge separation. Worth noting, the photocurrent response of CN is remarkably increased after the incorporation of Sr. Among all samples, the Pt/0.15Sr-CN sample exhibited the highest photocurrent signal, which is accredited to the fact, that Pt accepts the photo-induced electrons of 0.15Sr-CN and reduces its charge recombination rate. In addition, the electrochemical impedance spectroscopy (EIS) was also employed. Normally, the charge transfer resistance of the catalysts can be examined directly from the resultant arc radius of the Nyquist plots. Notably, smaller the arc radius, higher will be the charge separation in catalysts [52, 53]. The obtained Nyquist plots of the CN, 0.15Sr-CN, Au/0.15Sr-CN, and Pt/0.15Sr-CN samples are displayed in Fig. 8d. As obvious, the smallest arc radius is achieved for the Pt/0.15Sr-CN sample, suggesting the highest charge transfer and separation, and is in accord with the SPV, PL, and photocurrent density results.

### Photocatalytic Activities Evaluation

**Fig. 9:**
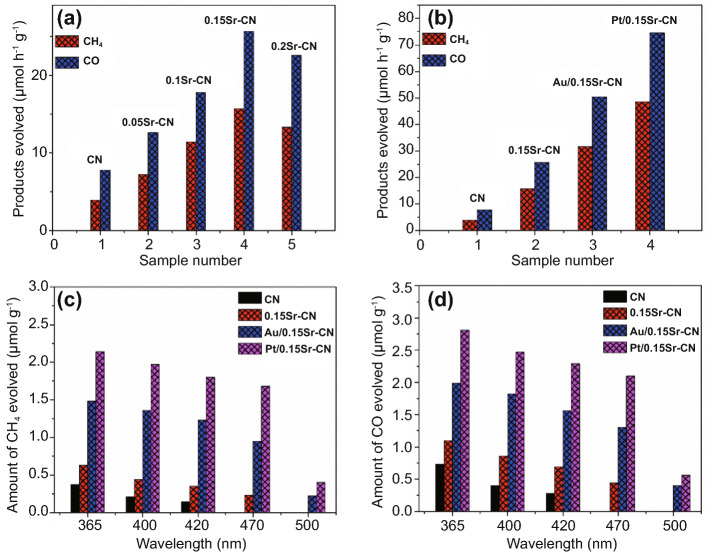
Visible light catalytic activities for CO_2_ conversion **a** over the CN and xSr-CN samples and **b** over the CN, 0.15Sr-CN, Au/0.15Sr-CN and Pt/0.15Sr-CN. Amount of CH_4_ produced (**c**), and amount of CO produced (**d**), over the CN, 0.15Sr-CN, Au/0.15Sr-CN and Pt/0.15Sr-CN under single wavelength photocatalytic experiments

The visible light catalytic-activity of the catalysts was explored by the reduction of CO_2_ as shown in Fig. 9a, b. The CN displayed low reduction ability by producing 3.92 μmol h^−1^ g^−1^ of CH_4_ and 7.78 μmol h^−1^ g^−1^ of CO gas. Notably, after Sr-incorporation, the photocatalytic CO_2_ reduction ability of CN is improved to a great extent. The optimized 0.15Sr-CN sample produced 15.7 μmol h^−1^ g^−1^ of CH_4_ and 25.64 μmol h^−1^ g^−1^ of CO. The CO_2_ conversion ability over the Au/0.15Sr-CN sample was obvious i.e., it produced 31.62 μmol h^−1^ g^−1^ of CH_4_ and 50.43 μmol h^−1^ g^−1^ of CO. The Pt/0.15Sr-CN sample exhibited the best photocatalytic activity with CH_4_ and CO yield of ~ 48.55 and ~ 74.54 μmol h^−1^ g^−1^, respectively. To confirm the photocatalysts stability, photocatalytic recyclable tests under visible light irradiations were performed. Interestingly, the CH_4_ and CO production rates from CO_2_ conversion over the Au/0.15Sr-CN and Pt/0.15Sr-CN catalysts (Fig. S14a-d) did not change remarkably after 4-cycles, confirming excellent stability of the catalysts.

Further, the quantum efficiencies of CN, 0.15Sr-CN, Au/0.15Sr-CN, and Pt/0.15Sr-CN samples for CO_2_ conversion were measured at wavelength 420 nm as shown in supporting information. The calculated quantum efficiencies for CO_2_ conversion over the CN, 0.15Sr-CN, Au/0.15Sr-CN, and Pt/0.15Sr-CN samples are 0.85%, 1.58%, 2.65%, and 2.92%, respectively, which are comparatively higher from those of the previous reports (Table S3). Thus, this work would greatly contribute to the photocatalytic field of CO_2_ conversion to value added products.

In order to validate the CO_2_ photoreduction activities, the photocatalytic H_2_O reduction experiments over the CN, *x*Sr-CN, Au/0.15Sr-CN, and Pt/0.15Sr-CN catalysts were carried out under visible light irradiations with the aid of methanol as the sacrificial holes agent as shown in Fig. S15a, b. The control experiments (in absence of photocatalysts) were performed that resulted in no H_2_ evolution. As obvious, the H_2_ evolved over the pristine CN under visible light irradiations is quite low (i.e., ~ 7 μmol h^−1^ g^−1^), while, a remarkable enhancement in H_2_ evolution was observed for the optimized 0.15Sr-CN sample (i.e., ~ 56.8 μmol h^−1^ g^−1^). Worth noting, the Au/0.15Sr-CN sample produced ~ 157 μmol h^−1^ g^−1^ of H_2_ under visible light irradiations. A significant increase in the H_2_ production (i.e., ~ 362 μmol h^−1^ g^−1^) was observed for the Pt/0.15Sr-CN sample. The superior catalytic H_2_ production over the Pt/0.15Sr-CN sample could be accredited to extended optical absorption and high charge carrier’s separation efficiency. The photocatalytic stability of the Au/0.15Sr-CN and Pt/0.15Sr-CN catalysts was explored by the recyclable tests for H_2_ evolution. Figure S15c, d reveals that the rate of H_2_ evolution during 4-cycles (each cycle of 4-h duration) did not changed considerably, which further validates the superior photocatalytic stability of the catalysts.

To support the enhanced catalytic activities, photocatalytic experiments for RhB dye and 2,4-dichlorophenol (2,4-DCP) degradation were performed under visible light irradiations. As revealed in Fig. S16a, b, the RhB degradation over pristine CN was about 31% in 2 h. After Sr-incorporation, the RhB degradation activity is gradually enhanced with increasing the Sr-content, especially for the 0.15Sr-CN sample that degraded 47% of RhB in 2 h. Interestingly, the Au/0.15Sr-CN and Pt/0.15Sr-CN samples, respectively, degraded 82 and 91% of RhB in 2 h irradiation period. Likewise, the 2,4-DCP degradation rates over the samples under visible light irradiations for 2 h are shown in Fig. S16c, d. Actually, it is very hard to break the chlorine-carbon bonds of 2,4-DCP. Thus, a photocatalyst with strong oxidation ability is required for efficient 2,4-DCP degradation [54]. As obvious, the pristine CN and 0.15Sr-CN samples, respectively, degraded 25 and 38% of the 2,4-DCP pollutant. However, the 2,4-DCP degradation over the Au/0.15Sr-CN and Pt/0.15Sr-CN samples was drastically improved, i.e., 69 and 81%, respectively. Based on the overall experimental and theoretical results, it is demonstrated that Sr-incorporation and noble metal deposition can significantly improve the activity of pristine CN due to band gap narrowing via the Sr-incorporation and superior charge carrier’s separation via the SPR effect of noble metal nanoparticles.

### Mechanism Discussion

**Fig. 10:**
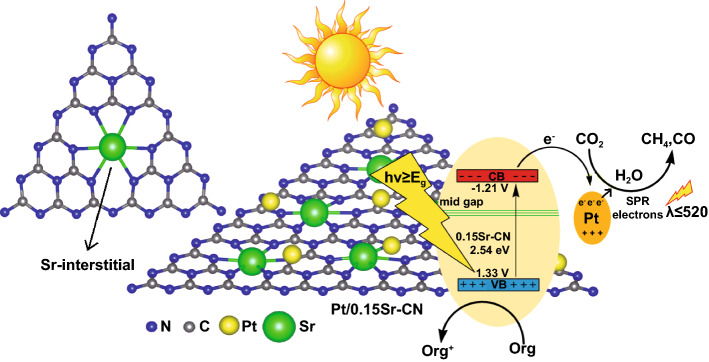
Schematic of the energy band-structure, charge separation and transfer and the surface redox reactions over the Pt/0.15Sr-CN catalyst

To investigate the role of Sr and noble metal nanoparticles in CO_2_ photoreduction, the CO_2_ conversion activity was performed under single wavelengths (365–500 nm) irradiation (2 h) as displayed in Fig. 9c, d. The CN displayed activity up to wavelength 420 nm. However, the 0.15Sr-CN sample displayed activity up to the wavelength 470 nm, which is due to the extended visible light absorption via the Sr-incorporation. These results reveal that the reduction ability of pristine CN and 0.15Sr-CN samples is still limited, and these materials are inappropriate for efficient visible light photocatalysis. The Au/0.15Sr-CN and Pt/0.15Sr-CN samples displayed little activities even at 500 nm wavelength, indicating that the noble metal nanoparticles deposition has remarkably extended the photo-activities of the catalysts toward visible light range due to the SPR effect. It is validated from the valance band XPS (Fig. S17) that the valance band edge of pristine CN is located at 1.4 V versus the NHE, which is also in agreement with the previous reports [55, 56]. On the other hand, the valance band edge of 0.15Sr-CN catalyst is slightly blue shifted and located at 1.33 V versus NHE. As validated via the UV–vis absorption and DFT results, the band gap of the optimized 0.15Sr-CN catalyst is reduced to 2.54, which accredits to the absorption wavelength of 488 nm. The conduction band edge of the catalyst could be predicted from the equation: E_CB_ = E_VB_ – E_g_ [57, 58], where E_CB_ is the CB potential, and E_g_ represents the band gap of photocatalyst. Hence, the predicted conduction band potential of 0.15Sr-CN catalyst versus NHE is -1.21 V. Based on the above considerations, we designed a schematic for charge transfer and separation in the optimized Pt/0.15Sr-CN photocatalyst as displayed in Fig. 10.

It is demonstrated that when noble metal (Pt or Au) nanoparticles are photodeposited onto the optimized 0.15Sr-CN catalyst surface, and the catalyst is exposed to visible light irradiations, charge carriers are generated. The excited-electrons of 0.15Sr-CN catalyst transfers to the Pt or Au surface where they would involve in CO_2_ reduction reactions. On the other side, the induced holes in the valence band of 0.15Sr-CN would involve in oxidation reactions. Thus, the surface redox ability of the photocatalyst would significantly improve. In other words, the significantly improved CO_2_ reduction activity of Pt decorated 0.15Sr-CN catalyst could be accredited to the enhanced optical absorption and superior charge separation via the band gap narrowing due to Sr-incorporation and to the SPR effect of noble metal nanoparticles.

## Conclusions

In summary, noble metal nanoparticles (Au and Pt) decorated Sr-incorporated pristine CN photocatalyst has successfully been fabricated via the simple calcination and photo-deposition methods. The photocatalysts were employed in photocatalysis for CO_2_ conversion under visible light irradiations. The Pt/0.15Sr-CN catalyst produced 48.55 and 74.54 μmol h^−1^ g^−1^ of CH_4_ and CO, respectively, and yield a quantum efficiency of 2.92%, much higher than that of the reported works so far, under the same experimental conditions. In addition, the long-term photocatalytic stability test reveals that the catalyst is highly stable and do not decompose during the photocatalytic process. The drastically improved photocatalytic performance of the photocatalyst is accredited to the extended solar light absorption and remarkably enhanced charge carrier’s separation via the Sr introduced mid gap states and the SPR effect induced by noble metal nanoparticles. This work will trigger the design of pristine CN-based high efficiency photocatalysts for solar fuel generation.

## Supplementary Information

Below is the link to the electronic supplementary material.

Supplementary file1 (PDF 2262 kb)
