# Supplementary material for: Plasmon Assisted Highly Efficient Visible Light Catalytic CO2 Reduction Over the Noble Metal Decorated Sr-Incorporated g-C3N4
Source: Nanomicro Lett. 2021 Oct 15;13:209. doi: 10.1007/s40820-021-00736-x (PMC8521553; doi:10.1007/s40820-021-00736-x)
Supplement: Supplementary file 1 — Supplementary file1 (PDF 2262 kb) [file 40820_2021_736_MOESM1_ESM.pdf]

Supporting Information for

Plasmon Assisted Highly Efficient Visible Light Catalytic CO_2 Reduction Over the Noble Metal Decorated Sr-Incorporated g-C_3N_4

Muhammad Humayun[1], Habib Ullah[2], Lang Shu[1], Xiang Ao[1], Asif Ali Tahir[2], Chungdong Wang[1, *], and Wei Luo[1, *]

[1]School of Optical and Electronic Information, Wuhan National Laboratory for Optoelectronics, Engineering Research Center for Functional Ceramics of the Ministry of Education, Huazhong University of Science and Technology, Wuhan 430074, P. R. China

[2]Environment and Sustainability Institute, University of Exeter, Penryn, TR10 9FE Cornwall, United Kingdom

*Corresponding authors. E-mails: apcdwang@hust.edu.cn (Chungdong Wang); luowei@mail.hust.edu.cn (Wei Luo)

Supplementary Figures and Tables

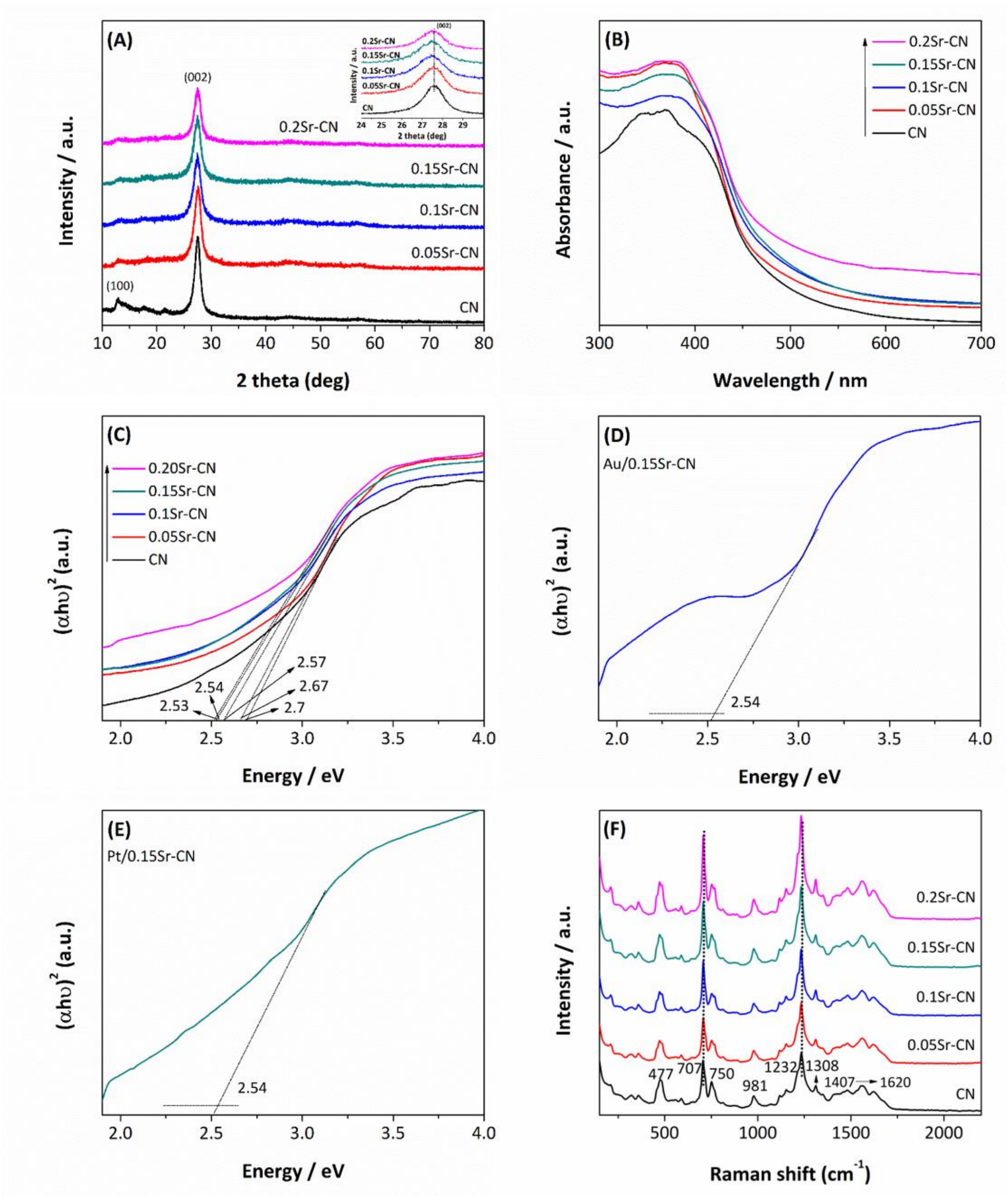

Fig. S1 XRD patterns (**a**), UV-vis absorption spectra (**b**) and the calculated energy band gaps (**c**) of CN and *x*Sr-CN samples. Calculated energy band gaps (**d**) of Au/0.15Sr-CN and (**e**) of Pt/0.15Sr-CN sample. Raman spectra of the CN and *x*Sr-CN samples (**f**)

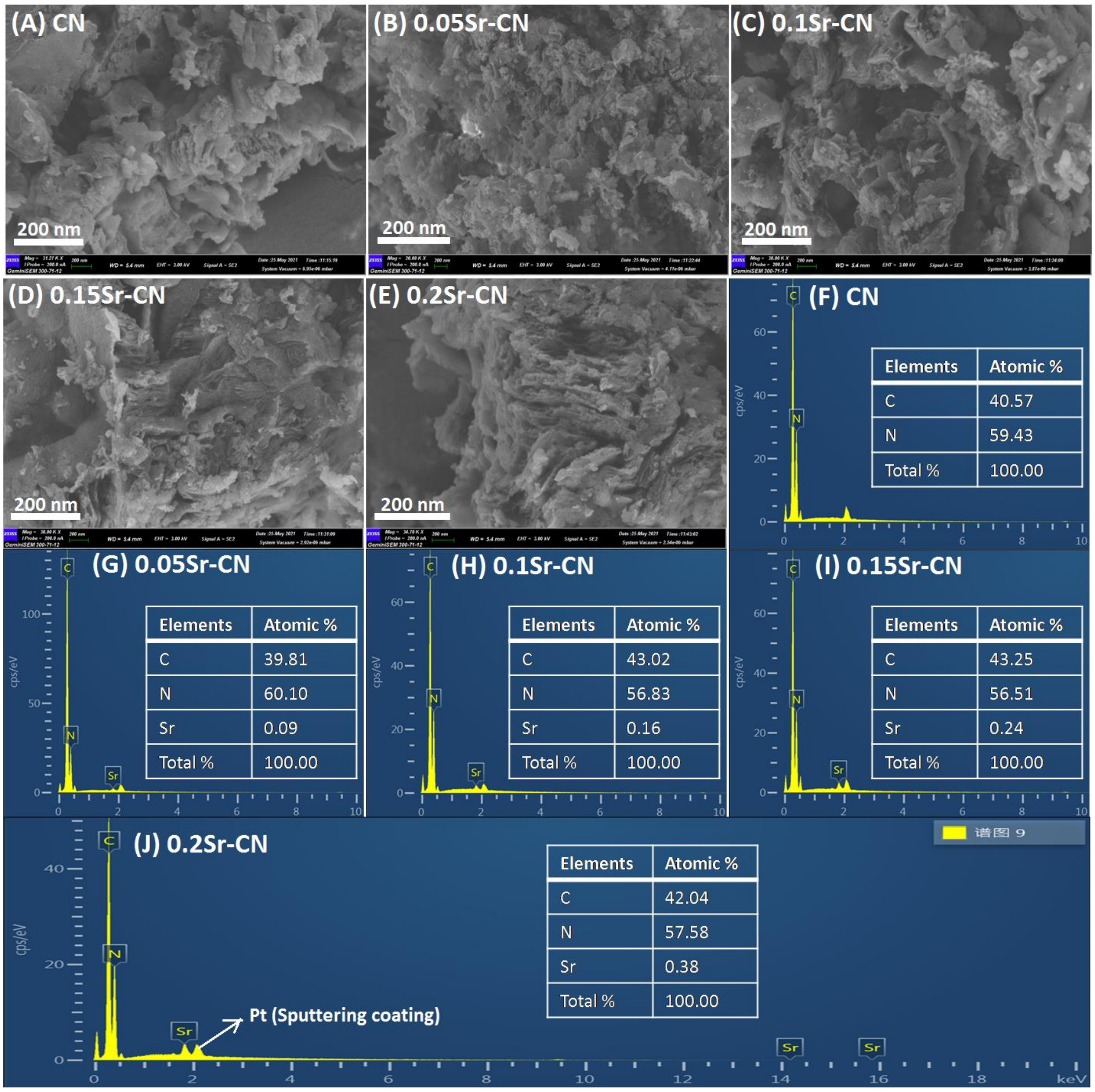

Fig. S2 Scanning electron microscopy (SEM) micrographs (**a**) of CN, (**b**) of 0.05Sr-CN, (**c**) of 0.1Sr-CN, (**d**) of 0.15Sr-CN and (**e**) of 0.2Sr-CN. Energy dispersive X-ray spectroscopy (EDX) spectra: (**f**) of CN, (**g**) of 0.05Sr-CN, (**h**) of 0.1Sr-CN, (**i**) of 0.15Sr-CN and (**j**) of 0.2Sr-CN samples with atomic percentage of each element as inset

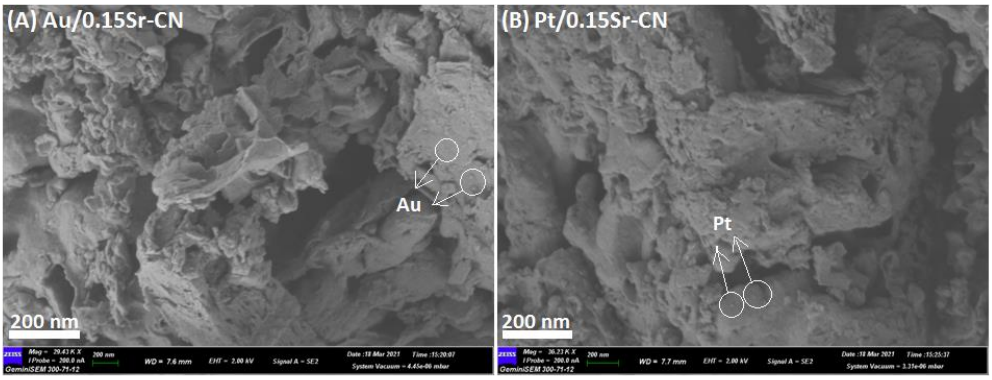

Fig. S3 SEM micrographs; (**a**) of Au/0.15Sr-CN, and (**b**) of Pt/0.15Sr-CN samples

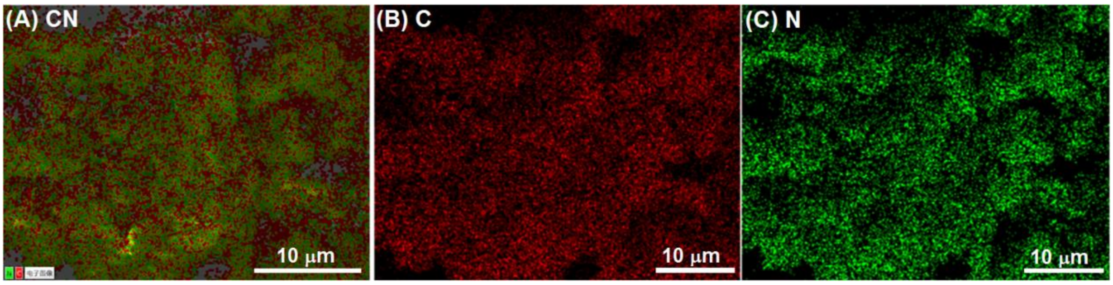

Fig. S4 Energy Dispersive X-ray Spectra (EDS) mappings of (**a**) CN, (**b**) C element and (**c**) N element

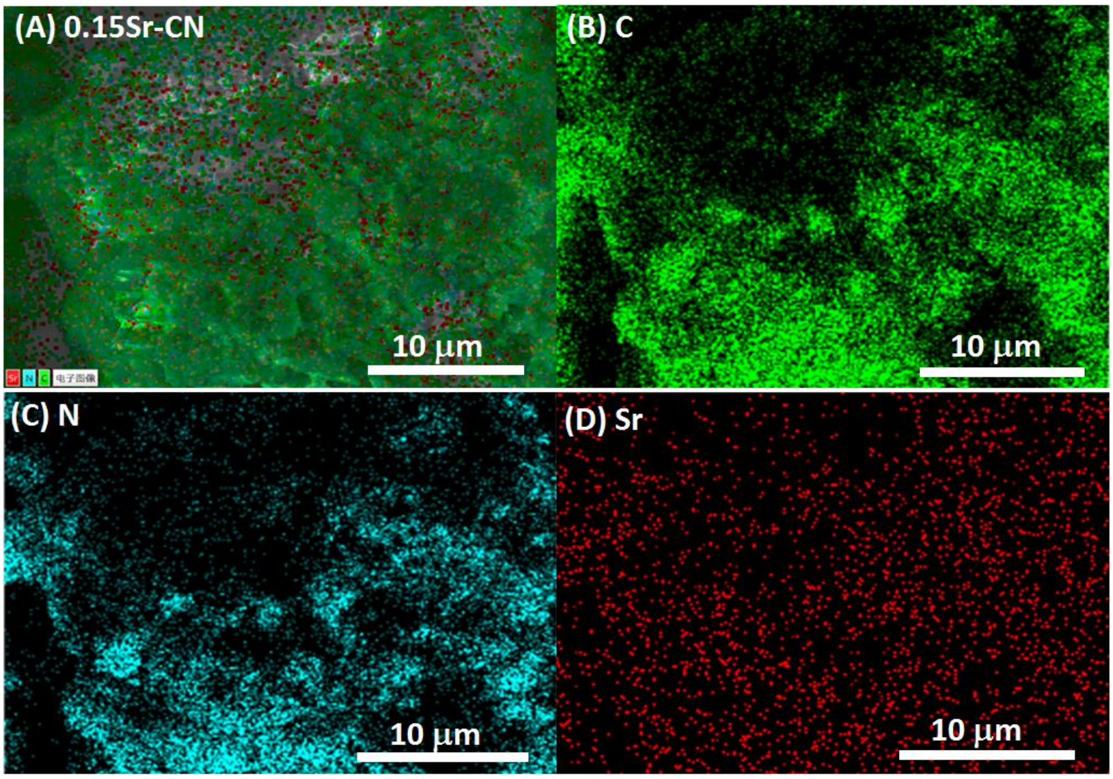

Fig. S5 Energy Dispersive X-ray Spectra (EDS) mappings of (**a**) 0.15Sr-CN, (**b**) C element, (**c**) N element and (**d**) Sr element

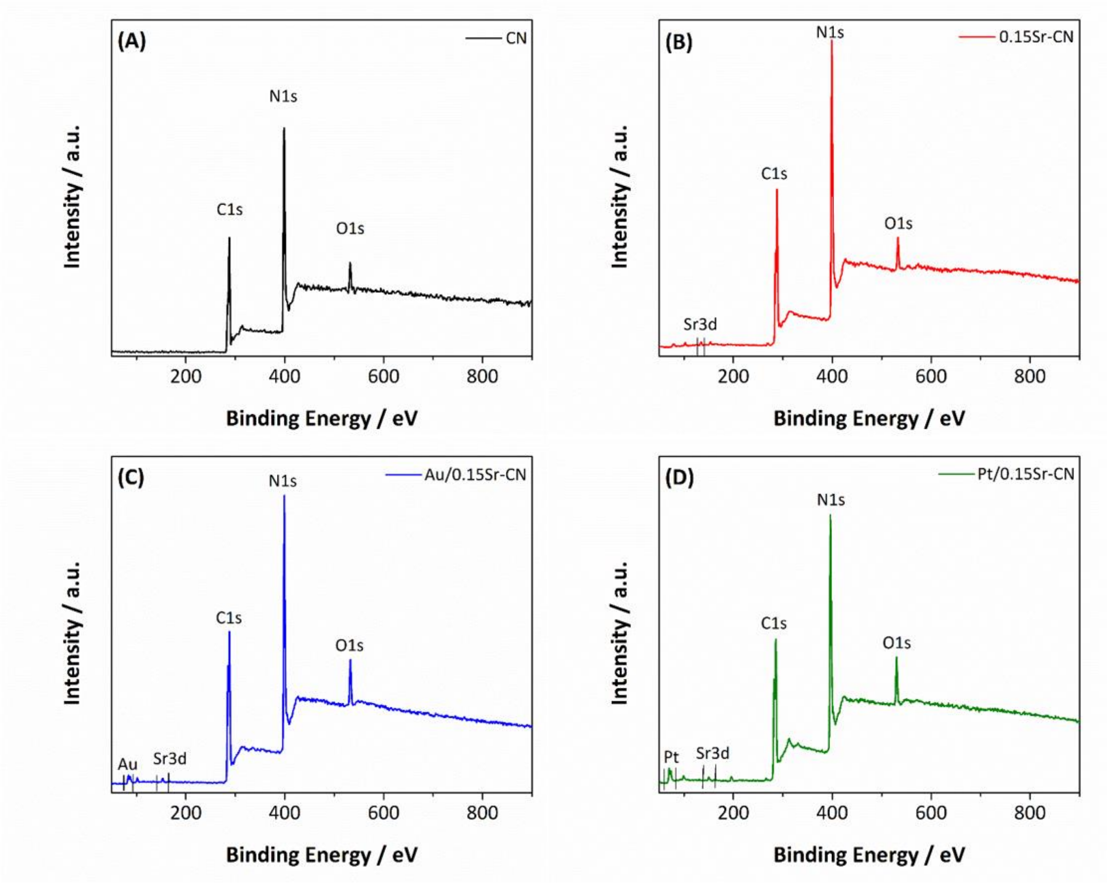

Fig. S6 XPS survey spectra (**a**) of CN, (**b**) of 0.15Sr-CN, (**c**) of Au/0.15Sr-CN and (**d**) of Pt/0.15Sr-CN

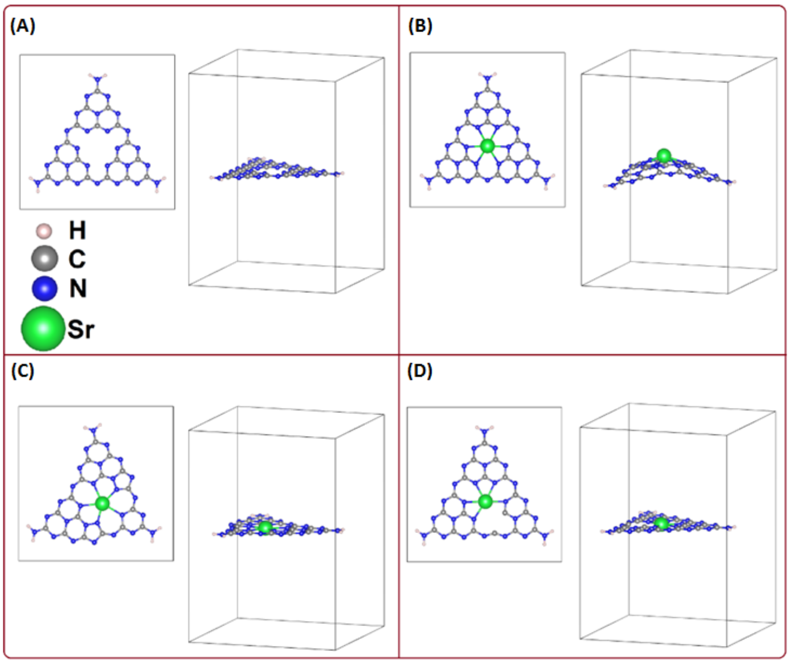

Fig. S7 Optimized relaxed structure of CN (**a**), 0.15Sr-CN (Interstitial site) doping (**b**), 0.15Sr-CN (C-substituted) doping (**c**), and 0.15Sr-CN (N-substituted) doping (**d**)

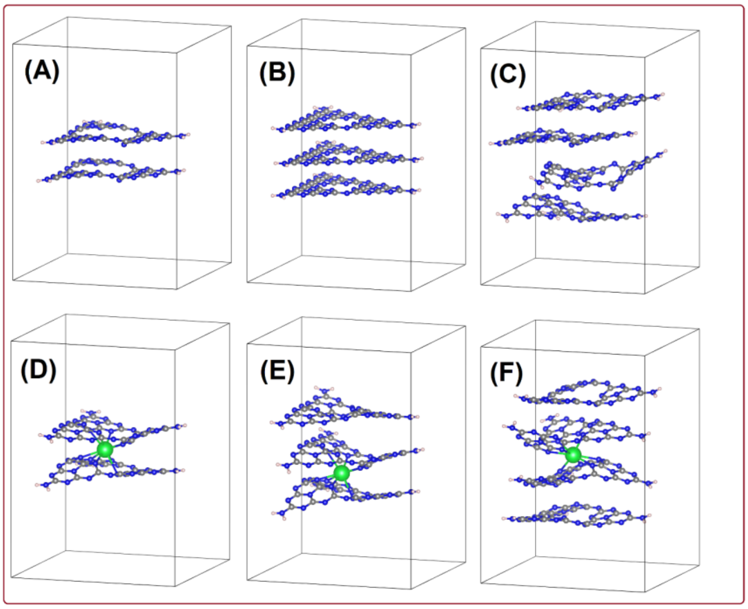

Fig. S8 The optimized relaxed structure of two layers (**a**), three layers (**b**), four layers (**c**), and Sr-incorporated two layers (**d**), Sr-incorporated three layers (**e**), and Sr-incorporated four layers of CN (**f**)

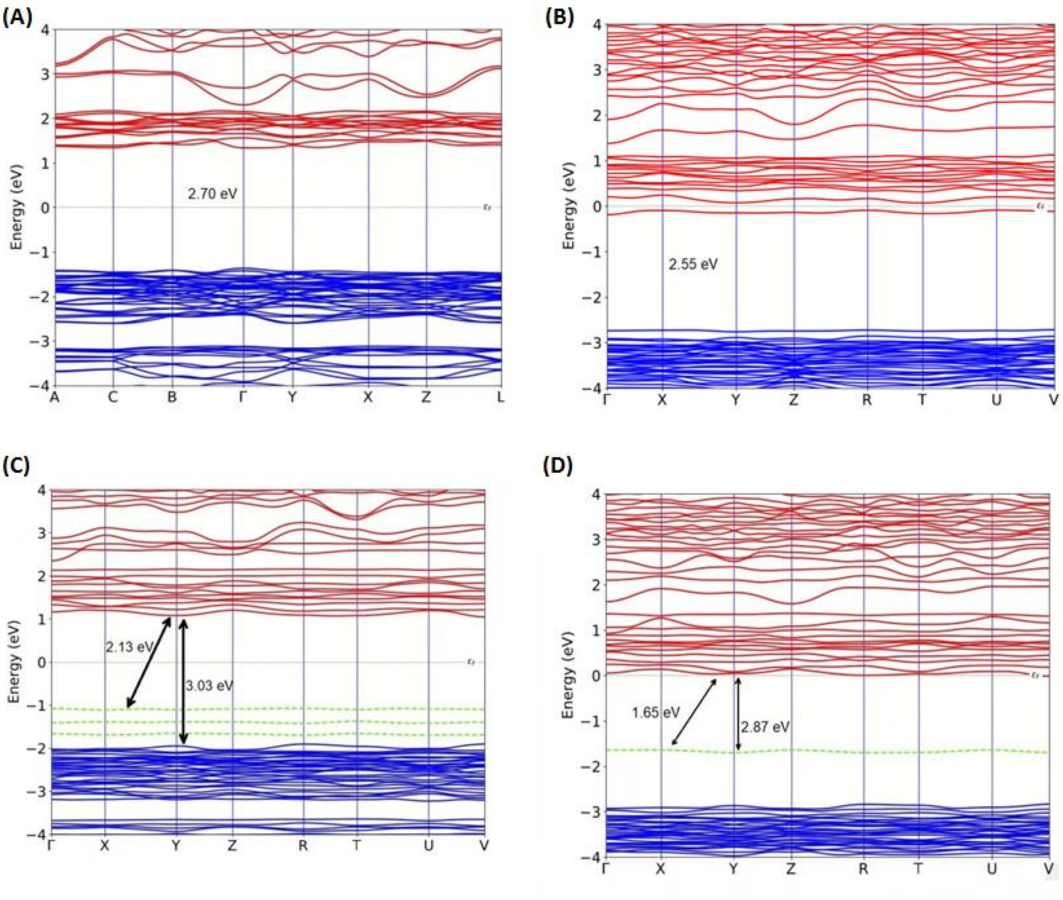

Fig. S9 DFT calculated band structures of CN (**a**), of 0.15Sr-CN (Interstitial site) doping (**b**), of 0.15Sr-CN (C-substituted) doping (**c**), and of 0.15Sr-CN (N-substituted) doping (**d**)

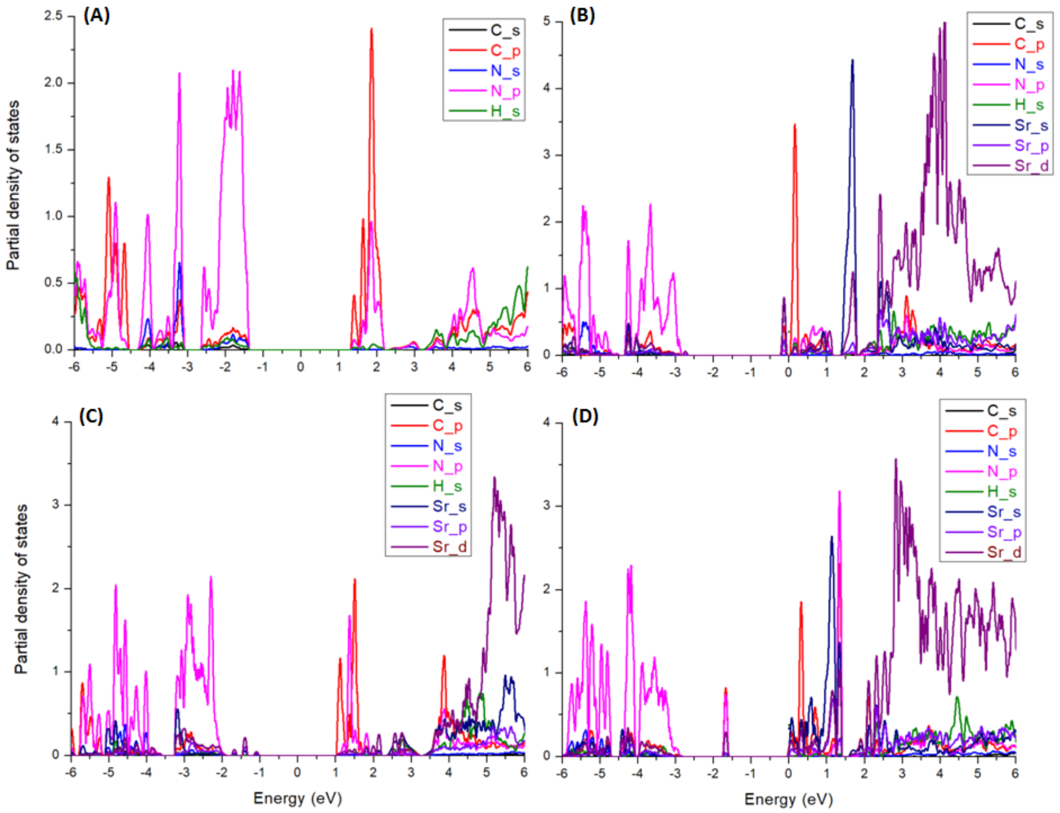

Fig. S10 PDOS plots of pristine CN (**a**), 0.15Sr-CN (Interstitial site) doping (**b**), 0.15Sr-CN (C-substituted) doping (**c**), and 0.15Sr-CN (N-substituted) doping (**d**)

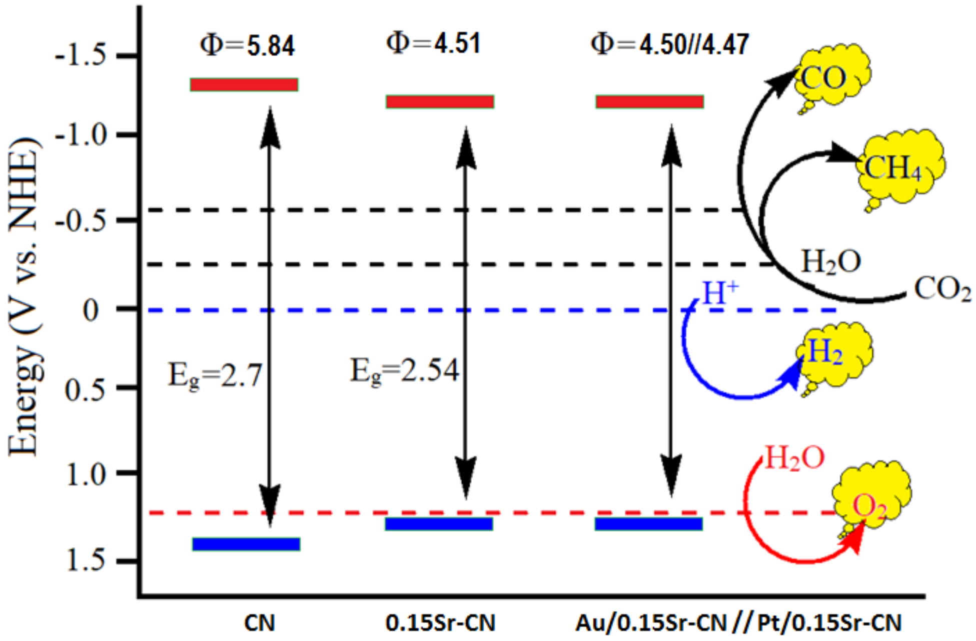

Fig. S11 Energy band diagrams of the pristine CN, 0.15Sr-CN (interstitial), and Pt and Au deposited 0.15Sr-CN

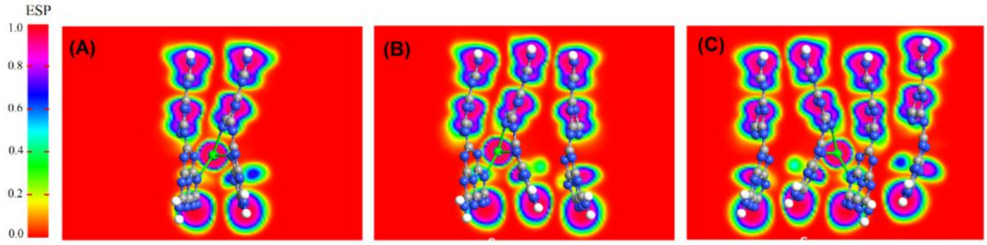

Fig. S12 Average electron density difference map for Sr-incorporated two layers (**a**), three layers (**b**), and four layers of CN (**c**)

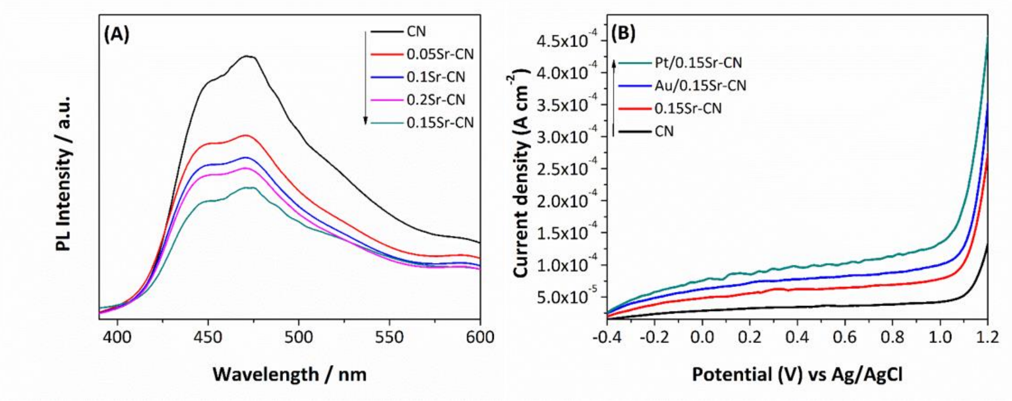

Fig. S13 Photoluminescence (PL) spectra of CN and *x*Sr-CN samples (**a**), and photoelectrochemical *I-V* curves of CN, 0.15Sr-CN, Au/0.15Sr-CN and Pt/0.15Sr-CN samples (**b**)

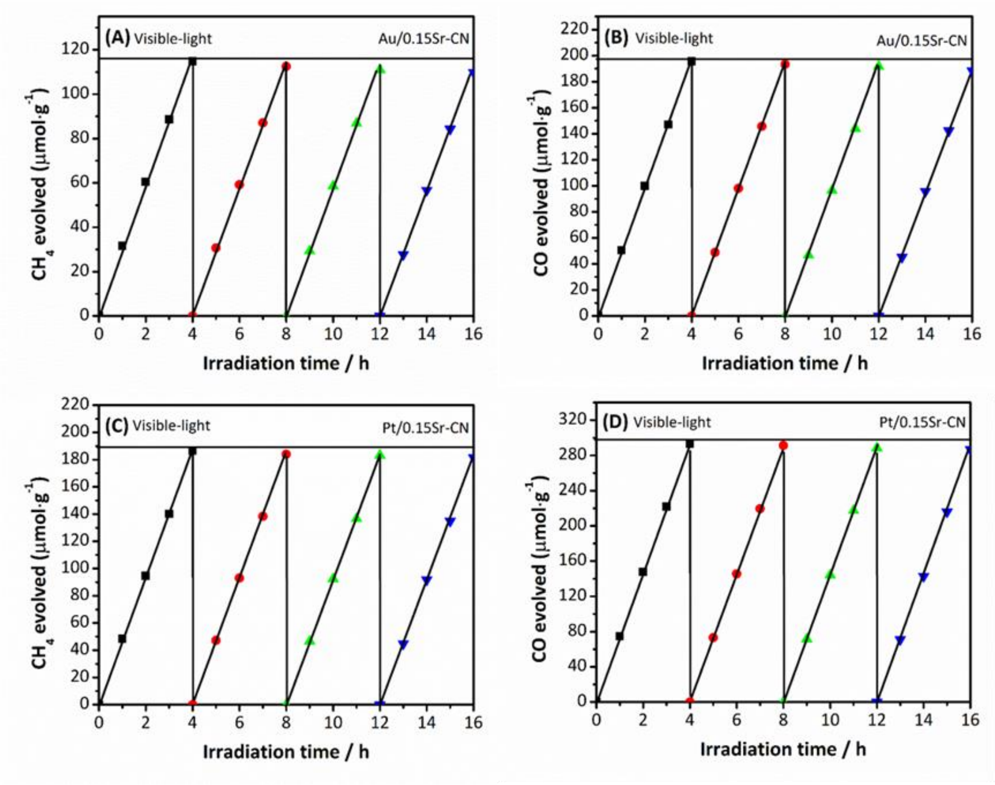

Fig. S14 Photocatalytic recyclable tests for CO_2 conversion to CH_4 and CO products (**a, b**) over the Au/0.15Sr-CN catalyst and (**c, d**) over the Pt/0.15Sr-CN catalyst

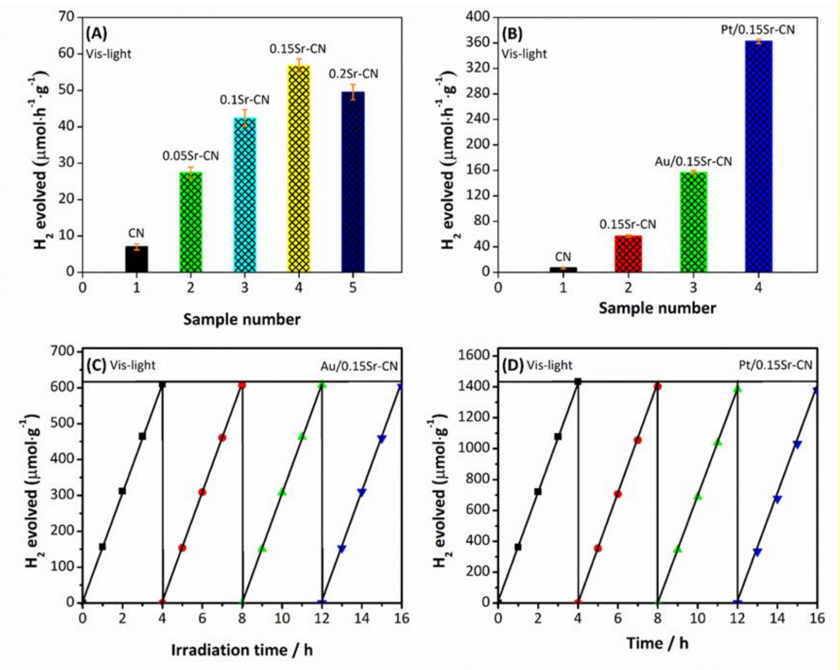

Fig. S15 Visible light catalytic activities for H_2 evolution (**a**) of CN and *x*Sr-CN samples (**b**) of CN, 0.15Sr-CN, Au/0.15Sr-CN and Pt/0.15Sr-CN samples. Photocatalytic recyclable tests for H_2 evolution (**c**) over the Au/0.15Sr-CN catalyst and (**d**) over the Pt/0.15Sr-CN catalyst

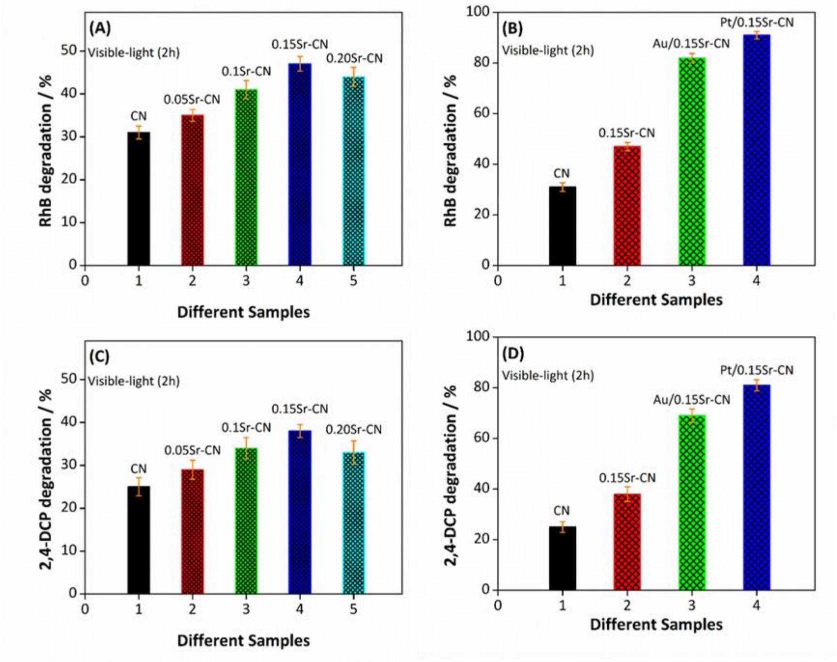

Fig. S16 Visible light catalytic activities for RhB dye degradation (**a**) over CN and *x*Sr-CN samples and (**b**) over CN, 0.15Sr-CN, Au/0.15Sr-CN and Pt/0.15Sr-CN samples. Visible light catalytic activities for 2,4-DCP degradation (**c**) over CN and *x*Sr-CN samples and (**d**) over CN, 0.15Sr-CN, Au/0.15Sr-CN and Pt/0.15Sr-CN samples

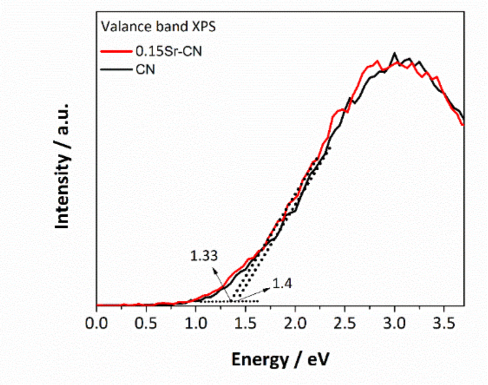

Fig. S17 Valence band XPS spectra of CN and 0.15Sr-CN samples

Table S1 Total free energies of pristine CN and Sr-doped CN species

Species	*Total free energies*
Pristine CN	-15602.50103
Sr-CN (Interstitial)	-16534.02436
Sr-CN (C-substituted)	-16371.92395
Sr-CN (N-substituted)	-16251.95281

Table S2 Work function, VBM, CBM, and band gap values in eV

Species	*Work functions*	*CBM*	*VBM*	*Bandgaps*
CN	5.84	-1.37	1.33	2.70
Sr-CN (Interstitial)	4.51	-2.74	-0.19	2.55
Sr-CN (C-substituted)	5.51	-1.08	1.05	2.13
Sr-CN (N-substituted)	4.73	-2.87	0	2.87

S2 Quantum Efficiency Calculations for CO_2 Conversion

The quantum efficiency of CN, 0.15Sr-CN, Au/0.15Sr-CN and Pt/0.15Sr-CN samples for CO_2 conversion was calculated at λ=420 nm. The samples were irradiated with a 300 W Xe-lamp for 8 h. The average incident irradiation was determined to be 2.01 mW cm^{-2} by the Newport (Oriel instrument USA model 91150V ser. No 391/0118) and area of the light collector part was 6.5 cm^2. The amount of CH_4 produced over the CN,

0.15Sr-CN, Au/0.15Sr-CN and Pt/0.15Sr-CN samples at λ=420 nm was 1.4, 2.6, 4.35, and 4.8 µmol, respectively. While, the amount of CO produced over the over the CN, 0.15Sr-CN, Au/0.15Sr-CN and Pt/0.15Sr-CN samples under same wavelength was 1.78, 3.85, 5.65 and 6.5 µmol, respectively. The calculations are given below.

Quantum efficiency calculation for CN, 0.15Sr-CN, Au/0.15Sr-CN and Pt/0.15Sr-CN photocatalysts at λ=420 nm: Number of incident photons (N) in 8 h over 6.5 cm^2 area:

$$\mathrm{N} = \frac{E\lambda}{hc} = \frac{2.01\times10^{-3}\times6.5\times420\times10^{-9}\times8\times3600}{6.626\times10^{-34}\times3\times10^{8}} = 7.9\times10^{20}$$

$$\mathrm{QE} = \frac{\{(2\times\mu mol\ of\ CO) + (8\times\mu mol\ of\ CH_4)\}\times\ Avogadro\ number}{the\ number\ of\ incident\ photons}\times100$$

$$\mathrm{QE_{CN}} = \frac{\{(2\times1.78\times10^{-6}) + (8\times1.4\times10^{-6})\}\ \times\ 6.02\times10^{23}}{7.9\times10^{20}}\times100 = 0.85\%$$

$$\mathrm{QE_{0.15Sr-CN}} = \frac{\{(2\times3.85\times10^{-6}) + (8\times2.6\times10^{-6})\}\ \times\ 6.02\times10^{23}}{7.9\times10^{20}}\times100 = 1.58\%$$

$$\mathrm{QE_{Au/0.15Sr-CN}} = \frac{\{(2\times5.65\times10^{-6}) + (8\times4.35\times10^{-6})\}\ \times\ 6.02\times10^{23}}{7.9\times10^{20}}\times100 = 2.65\%$$

$$\mathrm{QE_{Pt/0.15Sr-CN}} = \frac{\{(2\times6.5\times10^{-6}) + (8\times4.8\times10^{-6})\}\times\ 6.02\times10^{23}}{7.9\times10^{20}}\times100 = 2.92\%$$

Table S3 Comparison of our results for CO_2 conversion with the previous reports

S.#	Photocatalysts	Source of light	Wavelength used	Quantum efficiency (%)	Refs.
1	NiS_2 QDs-g-C_3N_4	300 Xe-lamp	420 nm	2.03%	Colloid. & Surf. A: Phys. & Eng. Asp. 600, 2020, 124912.
2	CdSe/P-CN	300 Xe-lamp	420 nm	2.57%	Appl. Catal., B: Environ. 270, 2020, 118867
3	SnO_2/B, P-gC_3N_4	300 Xe-lamp	420 nm	2.02%	Appl. Catal., B: Environ. 201, 2017, 486-494.
4	Pt/0.15Sr-CN	300 Xe-lamp	420 nm	2.92%	Current work
